# Supplementary material for: Evaluation of agricultural products e-commerce logistics service capabilities in Heilongjiang Province based on entropy weight TOPSIS method
Source: PLoS One. 2025 Jun 25;20(6):e0325532. doi: 10.1371/journal.pone.0325532 (PMC12192297; doi:10.1371/journal.pone.0325532)
Supplement: Attachment — Submitted filename: Response to Reviewers.pdf [file pone.0325532.s003.pdf]

major revisions

Evaluation of agricultural products e-commerce logistics service capabilities in Heilongjiang Province based on entropy weight TOPSIS method

To the Editor-in-Chief and Reviewers

We are honored to have the opportunity to submit the revised manuscript of the paper " Evaluation of E-commerce Logistics Service Capability of Agricultural Products in Heilongjiang Province Based on Entropy Weight TOPSIS Method" to PLOS for publication. We thank the editors and reviewers for their time and effort in providing feedback on our manuscript and for their insightful comments and valuable improvements on our paper. We have adopted most of the suggestions made by the reviewers. Please see below for a one-by-one response to the reviewers' comments and concerns. All section numbers refer to the revised manuscript file, with revision records attached .

Reviewer #1

The Reviewer's Comment #1.1

The precise research gap(s) and the major outcomes should be mentioned in the Abstract. Keywords need to be updated with standard keywords.

reply:

Thank you for the reviewer's valuable suggestions on the abstract. We will further clarify the research gaps and clearly present our main research results in the revision to ensure that the abstract can more accurately reflect the core contribution of the paper. At the same time, we will also update the keywords in the abstract according to the reviewer's suggestions, adopt more standard terms, and ensure that it conforms to the conventions in the field. Revisions on page 1

The Reviewer's Comment #1.2

The Introduction needs to be revised while specifying the research questions. Mentioning a number of articles without proper interlinks make this paper less interesting for readers.

reply:

Thank you for your valuable comments. We fully agree that the introduction should better link and integrate relevant literature to enhance the appeal of the article. During the revision process, we will reduce direct citations to multiple literatures, ensure that each citation has a clear logical connection, and can better support the research questions. We believe that this will improve the readability and overall quality of the article. Thank you again for your careful review and constructive feedback. The revised content is on pages [2-4]

The Reviewer's Comment #1.3

Citation of multiple references must be avoided.

reply:

Thank you for your valuable suggestions. We fully understand the importance of avoiding too many references in the introduction, so as not to affect the fluency and appeal of the article. During the revision process, we will ensure that only the most relevant and critical literature is cited , and avoid excessive citations, striving to find a balance between conciseness and completeness. Thank you for your careful review, and we will make the revisions according to your suggestions. The revisions are on pages [2-4]

The Reviewer's Comment #1.4

Sect 2 describes Operational capability, Logistics carrying capacity etc., the majority of which consists of existing knowledge. Thus, these can be moved to appendix along with proper referencing.

reply:

Thank you for your valuable suggestions on Section 2. We agree that this section contains a lot of existing knowledge and has little impact on the core contribution of the study. Therefore, we will move this section and related references to Appendix 1 as you suggested, so as not to affect the core focus of the article and make the article structure more concise and clear. Thank you again for your careful review and constructive comments.

The Reviewer’s Comment #1.5

Sect 3 should be improved with more details. Also, Sect 4.1.1 can be moved to appendix.

reply:

Thank you for your constructive comments on Sections 3 and 4. We will improve Section 3 based on your suggestions and provide more details to explain the relevant content more clearly. As for Section 4.1.1 of Section 4, we also agree that this part can be moved to Appendix 3 to make the article more concise while ensuring that readers can refer to the detailed information as needed. Thank you for your careful review and we will make the revisions according to your suggestions.

The revised content is on pages [6-9]

The Reviewer’s Comment #1.6

What is the source of data? Has the author followed the ethical practices? If yes, the ethical disclosure certificate must be enclosed as a supplemantary material.

reply :

Thank you for the reviewer's key questions. Regarding the source of the data, we will clearly state the source of the data in the revision and ensure its transparency. If there are any ethical issues involved in the research process, we will further explain that we have strictly followed the relevant ethical standards. If the reviewer believes that supplementary materials are needed, we will attach an ethical disclosure certificate as supplementary material to ensure compliance and transparency. Thank you again for your careful review and valuable suggestions. The revision is in Section 4, page 10

The Reviewer’s Comment #1.7

Authors need to provide further analysis, in addition to those in Table 4.19. Describe on calculation of entropy value e and information utility value d of each item.

reply :

Thank you for your valuable suggestions on Table 4.19. We will further provide the calculation process of the entropy value and information utility value d for each item according to your request, and describe these calculation steps in detail in the revision. This will help readers better understand the basis and process of data analysis. Thank you for your careful review, we will make revisions based on your suggestions. The revisions are on pages [14-15, 16-17, 18, 21-22, 25]

The Reviewer's Comment #1.8

There are some typos and language quality should be improved.

reply :

Thank you for your feedback on the language quality. We have carefully checked the entire article and will correct all spelling errors. In addition, we will further optimize the language of the article to improve its overall quality and fluency. Thank you for your careful review, and we will ensure that the article reaches a higher standard in terms of language expression. Thank you again for your careful review and valuable suggestions.

Reviewer #2

The Reviewer's Comment #2.1

The explanation of the entropy weight-TOPSIS method, while detailed, can benefit from a simplified flow diagram or visual representation to enhance clarity for broader audiences.

reply:

Thank you for the reviewer's valuable comments on our work. We understand that the explanation of the entropy weight -TOPSIS method is relatively detailed. In order to make it easier for a wider range of people to understand, we will add a simplified flowchart or visual representation based on your suggestion to improve the appeal and understandability of this section. We will include this improvement in the revised version and sincerely thank you for your suggestions and support. The simplified flowchart is added in Appendix 2

The Reviewer’s Comment #2.2

The manuscript mentions the use of SPSS to handle missing data but lacks details about the imputation method used. Please elaborate on how the missing values were filled and their potential impact on the results.

reply:

Thank you for the reviewer's valuable comments on this paper. Regarding the part about using SPSS to handle missing data, we did not provide enough details in the article. We will explain in detail the imputation method used in the revised version, including the specific imputation technique (such as mean imputation, recovery imputation or other methods), and the reasons why we chose this method. In addition, we fill in the discussion of the potential impact of the missing data on the results and evaluate the validity and effectiveness of this imputation on the analysis results. Thank you for your suggestions, and we will further improve this part in the revised version.

Revisions in Section 4, page 13

The Reviewer’s Comment #2.3

The discussion section could expand on why Heilongjiang underperforms relative to coastal provinces. Highlight specific challenges such as infrastructure, policies, or market dynamics.

reply:

Thank you for the reviewers' valuable comments on our paper. Regarding the Discussion section, we agree that we should delve deeper into specific challenges when analyzing why Heilongjiang has recently lagged behind the coastal provinces. Based on your suggestions, we will expand the Discussion section to focus on specific issues in infrastructure, policies, or market dynamics, and explore how these factors affect Heilongjiang's development. We believe that this supplement provides a more in-depth additional background analysis and further enriches the content of the paper. Thank you for your valuable suggestions, we will carry out and modify this section in the revised version. The revisions are in Section 5, pages 49-50

The Reviewer’s Comment #2.4

The countermeasures suggested in the conclusion are general. Adding concrete steps, like specific government initiatives or partnerships, would strengthen the practical applicability of the recommendations.

reply:

Based on your suggestions, we will add more specific steps and practical operation plans in the revised version, especially specific suggestions for urgent or possible partners of the government. We will describe how to implement these measures to improve their practicality, applicability and operability. Thank you for your valuable comments. We believe that our revised conclusion is more pragmatic and instructive. The revisions are in Section 6, pages 54-60.

The Reviewer's Comment #2.5

Some figures (e.g., Figure 4.5) lack detailed labels or legends, making it hard to interpret them without referring to the text. Consider enhancing their readability.

reply:

We thank the reviewer for his valuable comments. Regarding the lack of detailed labels or legends for figures (such as Figure 4.5), we understand that this may be

We will understandably add detailed labels and legends in the revised version to explain the figures more clearly and ensure that readers can easily understand the information presented by the figures even without text. We appreciate your suggestion and will improve the reliability of these figures to improve the overall resolution and comprehensibility of the paper. The revised content is in Section 4, Figure 4.1 , page 16; Section 4, Figure 4.2 , page 19; Section 4, Figure 4.3 , page 23; Section 4, Figure 4.4 , page 26; Section 4, Figure 4.1 , page 16

The Reviewer's Comment #2.6

Ensure references to studies from 2024 align with the journal's publication timeline. Future-dated references may need clarification or replacement with alternatives. Add the related article in references: K. Gao, T. Liu, Y. Rong et al., A novel BWM-Entropy-COPRAS group decision framework with spherical fuzzy information for digital supply chain partner selection, Complex & Intelligent Systems, 10 (2024), 6983-7008.

reply:

Thank you for your careful comments on our paper. Regarding the consistency of citations of the 2024 study with the journal publication schedule, we will carefully check and ensure that all cited references are consistent with the journal's publication timeline. If necessary, we will clarify references with future dates or replace them with alternative content. In addition, we will add relevant related articles to the references based on your suggestions, including:

K. Gao, T. Liu, Y. Rong, et al., "A novel BWM-Entropy-COPRAS group decision framework with spherical fuzzy information for digital supply chain partner selection," Complex and Intelligent Systems, 10 (2024), 6983-7008.

M. Aggarwal, R. Krishankumar, K. S. Ravichandran, et al., "Assessing the potential of organizations using fuzzy entropy ," Operations Research Forum, 4 (2023), 11, https://doi.org/10.1007/s43069-022-00178-0.

Cluster head selection in wireless sensor networks under uncertainty based on entropy- weighted TOPSIS," Telecommunications, 4 (2023), 678-692.

A. Hussain, K. Ullah, et al., "Energy supplier selection based on TOPSIS method using new ideas of complex fuzzy rough information for multi-attribute decision making," Energy Strategy Review, 54 (2024), 101442, https://doi.org/10.1016/j.esr.2024.101442.

Z. Ali, Z. A. Khan, et al., "Frank-based TOPSIS methodology based on intuitive linguistic aggregation operator and its applications," IEEE Access, 12 (2024), 91605-91618.

We thank you for your valuable suggestions and will make relevant adjustments and additions in the revised

version according to your requirements. The revised content is in the references, pages 65-69

The Reviewer’s Comment #2.7

The abstract can be condensed slightly to focus more on key findings and their significance while trimming excessive methodological detail.

reply:

Thank you for your valuable comments on the abstract of our paper. We understand that the abstract should be more concise and clear, focusing on key findings and their importance. According to your suggestions, we will appropriately compress the content of the abstract, reduce the detailed description of the method section, and focus more on the main findings of the study and its academic and practical significance. This will make the abstract more concise and help readers quickly grasp the core content of the study. Thank you again for your suggestions, and we will make corresponding adjustments in the revised version.

The Reviewer’s Comment #2..8

While the manuscript is generally well-written, minor grammatical corrections and phrasing adjustments are needed in sections like the introduction (e.g., repetitive use of "e-commerce logistics service capabilities").

reply:

Thank you for your overall evaluation of our manuscript and your valuable comments on grammar and wording. We will carefully check the grammatical issues in the introduction and other parts according to your suggestions, and adjust the repeated terms (such as "e-commerce logistics service capabilities") to ensure more accurate and fluent expression. We will make corresponding corrections and adjustments in the revised version. Thank you for your careful suggestions to help us improve the quality of the paper.
