# Supplementary material for: Evaluation of agricultural products e-commerce logistics service capabilities in Heilongjiang Province based on entropy weight TOPSIS method
Source: PLoS One. 2025 Jun 25;20(6):e0325532. doi: 10.1371/journal.pone.0325532 (PMC12192297; doi:10.1371/journal.pone.0325532)
Supplement: Attachment — Submitted filename: Response to Reviewer.pdf [file pone.0325532.s004.pdf]

Minor Revision Edited Reply

Evaluation of agricultural products e-commerce logistics service capabilities in Heilongjiang Province based on entropy weight TOPSIS method

To the Editor-in-Chief and Reviewers

We are honored to have the opportunity to submit the revised manuscript of the paper " Evaluation of E-commerce Logistics Service Capability of Agricultural Products in Heilongjiang Province Based on Entropy Weight TOPSIS Method" to PLOS for publication. We thank the editors and reviewers for their time and effort in providing feedback on our manuscript and for their insightful comments and valuable improvements on our paper. We have adopted most of the suggestions made by the reviewers. Please see below for a one-by-one response to the reviewers' comments and concerns. All section numbers refer to the revised manuscript file, with revision records attached .

Reviewer # 4

The Reviewer's Comment #4.1

An abstract should address these questions: what are you trying to do, why, what you found and what is the significance of your findings. Rewrite and improve.

reply:

Thank you to the reviewers for their valuable suggestions on the abstract. We have adjusted and optimized the abstract according to the reviewers' requirements to ensure that the purpose, significance, main findings and academic and practical value of these findings are more clearly answered. The revised abstract is more compact, clearly points out the background and motivation of the study, and clearly presents the main findings of the evaluation of agricultural product e-commerce logistics capabilities in Heilongjiang Province and 31 provinces and cities across the country by constructing a logistics service capability evaluation index system and using the entropy weight-TOPSIS method. In addition, the abstract also highlights the practical significance of the research results, clearly puts forward policy recommendations for cold chain logistics, urban and rural logistics layout and digital transformation, and emphasizes the value of this study in improving the efficiency of agricultural product e-commerce logistics in Heilongjiang Province and exploring high-quality development paths in other major agricultural production provinces. Thank you again for your careful review and constructive feedback. The revised content is on pages [1-2]

The Reviewer's Comment #4.2

There are several studies already developed in the literature. What is the utility of your proposed study is better than previous.

reply:

We thank the reviewers for their valuable comments on our study. We strongly agree with the reviewer's point that most studies in the existing literature focus on the macro level and usually use static cross-sectional data for comparison, lacking a systematic description of regional dynamic evolution, especially for important agricultural production areas such as Heilongjiang . To this end, this study has achieved breakthroughs and improvements in the following aspects compared with previous studies:

First, based on the ten-year panel data of Heilongjiang Province from 2013 to 2022, and combined with the horizontal data of 31 provinces and cities across the country in 2022, we constructed a comprehensive evaluation system that integrates vertical and horizontal analysis. Compared with previous static horizontal comparisons, this study not only dynamically evaluates the development trajectory of Heilongjiang Province's logistics service capabilities, but also reveals its relative ranking and structural shortcomings nationwide, significantly enhancing the timeliness and comparative value of the research.

Secondly, this paper is more detailed in the construction of the index system, which fits the actual situation of e-commerce logistics. Unlike previous studies that mainly focus on basic indicators such as transportation costs and delivery time, we focus on logistics carrying capacity, technological innovation capabilities, operational capabilities and economic development capabilities in the first-level dimension, and refine them into 12 third-level indicators, covering the core elements of modern e-commerce logistics such as cold chain coverage, intelligent warehousing, and information system construction, thereby improving the comprehensiveness and scientificity of the evaluation.

In terms of methodology, this paper introduces the entropy weight -TOPSIS combined model to overcome the bias risk brought by the traditional subjective weighting method. At the same time, we handle missing values through multiple interpolation methods to ensure the continuity and reliability of panel data evaluation, which is better than the common simple mean method or deletion method.

In addition, this article focuses specifically on Heilongjiang, a typical region with weak logistics infrastructure and sparse population. Through the traceability analysis and horizontal comparison of the causes of fluctuations in logistics capacity, it accurately identifies the key bottlenecks that restrict development, such as the lagging construction of cold chain systems, insufficient digital capabilities, and inefficient regional distribution. It also

puts forward more feasible policy recommendations, such as promoting the integration of "digital + logistics", building a regional collaborative distribution network, and developing a localized e-commerce platform, which significantly enhances the practical pertinence and application value of the research.

Therefore, compared with previous studies, this paper has achieved important innovations in research perspective, data depth, indicator structure and policy guidance, and has provided a more scientific, systematic and promotional theoretical and practical path for improving the e-commerce logistics service capabilities of agricultural products in major agricultural production areas.

Thank you again for your careful review and constructive feedback. The specific revisions are on pages [3-4]

The Reviewer’s Comment #4.3

Comparative discussion needs further explanation. Please do more work on it.

reply:

We thank the reviewers for their valuable suggestions on our research. In response to the reviewers' comments on the need for further explanation in the comparative discussion section, we have made further expansion and revisions to more clearly demonstrate the gap between Heilongjiang Province and other developed regions in terms of agricultural product e-commerce logistics service capabilities and emphasize the bottlenecks and challenges of its development.

In the section on transportation network limitations, we further analyzed Heilongjiang Province’s deficiencies in logistics costs, cold chain logistics systems, etc., and made detailed comparisons with developed regions such as the Yangtze River Delta and the Pearl River Delta. By further elaborating on the limitations of Heilongjiang’s geographical location and transportation infrastructure compared to economically developed coastal regions, especially the gap in cold chain logistics systems and logistics timeliness, we explained in detail how these limitations affect the market competitiveness of Heilongjiang Province’s agricultural product e-commerce.

In the weak part of warehousing and distribution system, we supplemented the discussion on the limitations of modern warehousing facilities, intelligent sorting technology and terminal distribution network in Heilongjiang Province, and compared it with the widespread application of intelligent warehousing and distribution technology in the Yangtze River Delta region. We further elaborated on the shortcomings of Heilongjiang in terms of warehousing facility layout, degree of intelligence and distribution network coverage, especially the inefficiency faced in the "manual workshop" stage with a large reliance on manpower.

In the part about the relative lack of policy support, we discussed the limitations of Heilongjiang's policy

environment in more detail, and pointed out that compared with coastal provinces, Heilongjiang's policy support and incentives are still in the exploratory stage, and a systematic and sustainable support mechanism has not been formed. At the same time, we also supplemented the successful experience of coastal provinces in supporting agricultural product e-commerce logistics policies, and explained in detail how Heilongjiang's shortcomings in policy incentives have restricted the rapid development of the e-commerce logistics industry.

We also expanded the gap between market demand and competitive environment and further analyzed the reasons for insufficient market demand and immature industry competition environment in Heilongjiang. By comparing the high-frequency characteristics of e-commerce penetration and market demand in coastal provinces, we explained in detail that due to the small market size, low rural e-commerce penetration rate and incomplete consumer e-commerce habits in Heilongjiang, logistics companies are not enthusiastic about infrastructure and technology investment.

Finally, in the part where the agglomeration effect of enterprises is not obvious, we further discussed that Heilongjiang lacks large-scale e-commerce logistics companies with industry-leading roles, resulting in an incomplete industrial chain, lack of innovation vitality and resource integration capabilities. By comparing the agglomeration effect of enterprises with that of coastal provinces, we emphasized the challenges that Heilongjiang Province faces in forming a complete industrial ecology and improving the overall logistics service level.

Through the above revisions, we further elaborated on the constraints of Heilongjiang Province in terms of e-commerce logistics service capabilities for agricultural products, and compared the gap between Heilongjiang Province and other developed regions in terms of infrastructure construction, policy support, market demand, enterprise agglomeration, etc. These supplements and expansions will help to more comprehensively demonstrate the current status and development bottlenecks of Heilongjiang Province's e-commerce logistics service capabilities, and provide a more detailed analytical basis for future policy formulation and industrial development.

for your careful review and constructive feedback. Specific revisions are on pages [41-47]

The Reviewer’s Comment #4.4

Please improve the conclusion section. Also, limitations in the developed approach should be discussed in the conclusions section.

reply:

We thank the reviewer for his valuable comments on our study. Based on the reviewer's suggestions, we have improved the conclusion section and added a discussion of the limitations of the research method to make the

research results more comprehensive.

In the conclusion, we summarize the results of the multi-dimensional evaluation of Heilongjiang Province's agricultural product e-commerce logistics service capabilities, and elaborate on the main findings in terms of logistics carrying capacity, technological innovation capability, operational capability, economic development capability, etc. Through further improvements, we have a deeper discussion of each dimension, highlighting the gap in logistics service capabilities between Heilongjiang Province and developed coastal provinces, especially in cold chain logistics, technological innovation, and supply chain management.

At the same time, according to the reviewers' suggestions, we added a discussion on the limitations of the research method in the conclusion section. Specifically, we pointed out the advantages of the entropy weight method and TOPSIS method in this study, and also emphasized their limitations, including the failure to consider the weight distribution of subjective factors and the assumption of independence of each indicator. In addition, we also mentioned that this method is a static analysis and cannot reflect the dynamic trend of logistics service capabilities. In terms of data, some indicators rely on government statistical yearbooks and public information. The lag of these data and the consistency of regional data may affect the comprehensiveness and accuracy of the results.

In order to address these limitations and promote future research, we suggest using structural equation models (SEM), dynamic panel models or spatial econometric methods to improve the ability to describe causal paths, spatiotemporal effects and regional differences. At the same time, tools such as subjective empowerment mechanisms and machine learning algorithms can be introduced to build a more scientific and flexible logistics service capability evaluation system, thereby improving the adaptability and practical value of the model and providing a deeper decision-making basis for regional logistics optimization.

We thank the reviewers for their insightful and constructive comments on our study. We believe that the quality and depth of the paper have been further improved through these revisions.

Thank you again for your careful review and constructive feedback. Specific revisions are on pages [47-51] [56]

The Reviewer's Comment #4.5

The notations used should be rechecked.

reply:

We thank the reviewer for pointing out the problems with the use of symbols. We have comprehensively sorted out and standardized the symbols and indicator expressions in the full text, focusing on checking the consistency

and accuracy of mathematical formulas, model expressions, indicator abbreviations and variable symbols to ensure that their use in the full text is consistent, clear in meaning, and in line with academic norms.

At the same time, we have also unified the descriptions of variable symbols in charts and formulas, and added explanations when they first appear to improve readability and logical clarity and avoid ambiguity in the reader's understanding process.

We thank the reviewers for their attention and reminder to this detail. We believe that these revisions will help improve the professionalism and standardization of the paper.

The Reviewer's Comment #4.6

The writing is recommended to be improved. The authors are suggested to proofread paper and restructuring of sentences are required for the entire manuscript.

reply :

We would like to thank the reviewers for their pertinent suggestions on the quality of the paper. We fully recognize the importance of clear and accurate language expression for academic communication. According to the reviewers' suggestions, we have comprehensively polished the language and optimized the sentence structure of the full paper, focusing on the following aspects:

Standardization of language expression : grammatical errors, incoherent sentences, repeated expressions and other problems in the original text were corrected to improve the academic and logical nature of the overall language;

Sentence structure optimization : some overly long or ambiguous sentences were restructured to make them more concise and clear, thus enhancing the clarity of information conveyed;

Accurate use of terminology : Unify and standardize professional terms, variable names and technical expressions to ensure that the terminology is used consistently and in accordance with the prevailing standards in the field;

English polishing and review : We also further proofread the full English text using professional language polishing tools/services to improve language fluency and readability.

We believe that through the above improvements, the overall expression quality of the paper has been significantly improved. We thank the reviewers for their strict requirements and constructive suggestions on our work.
