# Supplementary material for: Evaluation of agricultural products e-commerce logistics service capabilities in Heilongjiang Province based on entropy weight TOPSIS method
Source: PLoS One. 2025 Jun 25;20(6):e0325532. doi: 10.1371/journal.pone.0325532 (PMC12192297; doi:10.1371/journal.pone.0325532)
Supplement: Attachment — Submitted filename: Response_to_Reviewer_auresp_3.pdf [file pone.0325532.s005.pdf]

Minor Revision Edited Reply

Evaluation of agricultural products e-commerce logistics service capabilities in Heilongjiang Province based on entropy weight TOPSIS method

To the Editor-in-Chief and Reviewers

We are honored to have the opportunity to submit the revised manuscript of the paper " Evaluation of E-commerce Logistics Service Capability of Agricultural Products in Heilongjiang Province Based on Entropy Weight TOPSIS Method" to PLOS for publication. We thank the editors and reviewers for their time and effort in providing feedback on our manuscript and for their insightful comments and valuable improvements on our paper. We have adopted most of the suggestions made by the reviewers. Please see below for a one-by-one response to the reviewers' comments and concerns. All section numbers refer to the revised manuscript file, with revision records attached .

Reviewer

Journal Requirements: #

Please review your reference list to ensure that it is complete and correct. If you have cited papers that have been retracted, please include the rationale for doing so in the manuscript text, or remove these references and replace them with relevant current references. Any changes to the reference list should be mentioned in the rebuttal letter that accompanies your revised manuscript. If you need to cite a retracted article, indicate

reply:

We sincerely thank the Editor for the valuable reminder regarding the reference list. We have carefully reviewed all the references cited in our manuscript and confirm that none of them have been retracted. Therefore, no changes were required in this regard. Please do not hesitate to let us know if any further clarification is needed.

Additional Editor Comments:#

Dear author, before further process please rectify the following concenrs:The articles published in this journal should be written in English language. In some figure other languages are detected which make the confusion for the reader.

reply:

We sincerely thank the Editor for pointing out the issue regarding the presence of non-English text in some figures. In response to this valuable comment, we have thoroughly revised all relevant figures to ensure that they are entirely in English.

Specifically, the figures originally containing non-English content—namely Table 4.6 through Table 4.14—have now been fully translated into English. These revised tables are presented in the updated manuscript on pages 24–37.

We are grateful for your careful review and appreciate the opportunity to improve the clarity and quality of our submission.

Additional Editor Comments: #

Try to restuctured the section 6 as it is very long. You may consider to make different section.

reply:

We sincerely thank the Editor for the thoughtful suggestion regarding the structure and length of Section 6. In response, we have carefully revised this section to make it more concise and focused.

Specifically, we have reduced redundant content and streamlined the discussion to enhance clarity and readability, while retaining all essential information. These modifications can be found on pages 40–49 of the revised manuscript.

We believe the revised version improves the overall presentation of our findings, and we are grateful for the Editor's constructive feedback that guided this improvement.

Additional Editor Comments: #

Either use journal format to make the manuscript or should be consistant in writing with same font and size throughout the manuscript.

reply:

We sincerely thank the Editor for the helpful reminder regarding formatting consistency. In response, we have carefully revised the entire manuscript to ensure uniform formatting throughout. Specifically, the entire text has been formatted using Times New Roman font, size 9, as per the journal's formatting guidelines or for consistency. These adjustments have been applied across all sections of the manuscript to ensure a professional and cohesive

presentation. We appreciate the Editor’s attention to detail and the opportunity to improve the manuscript accordingly.
